# The relationship between social emotions and intuitive eating behaviors: an exploration based on text analysis

**DOI:** 10.3389/fpsyt.2025.1701751

**Published:** 2025-11-26

**Authors:** Jing Wu, Ranran Wang, Sihang Zhu, Tour Liu

**Affiliations:** 1 Faculty of Psychology, Tianjin Normal University, Tianjin, China; 2 Key Research Base of Humanities and Social Sciences of the Ministry of Education, Academy of Psychology and Behavior, Tianjin Normal University, Tianjin, China; 3 Tianjin Key Laboratory of Student Mental Health and Intelligence Assessment, Tianjin, China

**Keywords:** social media, emotional expression, eating behaviors, text mining, semantic network, LASSO regression

## Abstract

**Introduction:**

The present study provides novel insights into the psychological mechanisms linking social emotions and eating behaviors by integrating large-scale social media analysis with individual-level assessments.

**Methods:**

Two complementary approaches were employed: Study 1 analyzed 1,902 Weibo posts containing “diet” and “social” keywords through latent Dirichlet allocation topic modeling and semantic network analysis to identify thematic structures and interactional patterns; Study 2 surveyed 1,199 participants (aged 18–33) using the Intuitive Eating Scale and self-reported social situation texts, applying Linguistic Inquiry and Word Count (LIWC) and LASSO regression to identify language features of intuitive eating.

**Results:**

Study 1 revealed six psychological themes and a semantic framework connecting social–dietary interactions, health discourse, emotional states, and body image concerns, while Study 2 demonstrated that negatively valenced words (*e.g., sensitive, tiring*) were associated with lower intuitive eating, whereas positively valenced words (*e.g., relaxed, positive*) were associated with healthier eating patterns; moreover, negative emotion scores in social texts showed significant correlations with poorer intuitive eating (*p* < 0.05).

**Discussion/Conclusion:**

These findings illustrate associations between social emotional expression and eating behaviors, highlighting implications for emotion-sensitive interventions and the design of healthier online social environments.

## 1 Introduction

Eating is not merely a biological necessity but also a deeply psychological and social phenomenon. As a fundamental behavior for survival and adaptation, eating habits exert profound influence on both physiological and psychological functioning. Physiologically, a balanced diet sustains bodily homeostasis and promotes immune resilience, while imbalanced consumption—particularly of high-fat and high-sugar foods—can disrupt metabolic regulation and increase the risk of chronic conditions such as obesity and cardiovascular disease (1, 2). Psychologically, dietary quality plays a crucial role in mental well-being. Adherence to dietary patterns like the Mediterranean diet has been associated with lower risks of depression and anxiety, suggesting that food choices also serve as pathways to psychological regulation (3). Thus, eating behaviors reflect not only physical needs but also underlying psychological dynamics.

Notably, as inherently social beings, humans rarely make eating decisions in isolation. Eating often occurs within interpersonal contexts, where it functions not only to fulfill nutritional needs but also as a medium for social communication and emotional signaling (4, 5). Empirical research has revealed that social influences shape how much, what, and when people eat. Social norms, peer modeling, and group dynamics can alter food intake, particularly in contexts involving impression management or affiliation needs (6–8). For example, individuals may increase portion sizes when dining with close friends to express intimacy, yet restrict intake when eating with strangers to maintain self-control or social appropriateness (9, 10). Collectively, this evidence characterizes eating as a socially embedded practice that flexibly adapts to interpersonal contexts and relational demands.

Beyond these behavioral adjustments, interpersonal interactions inherently elicit social emotions — defined as emotions whose object is other human beings and social interaction, such as pride, embarrassment, guilt, and belonging (11). In other words, social emotions are feelings that arise within or are directed toward our relationships with others. They play a pivotal role in guiding social behavior, including eating decisions. Thus, eating can be conceptualized not only as a socially embedded practice but also as an emotionally mediated one, where social emotions serve as the psychological link between interpersonal dynamics and dietary choices. In this sense, eating decisions are fundamentally shaped by emotions arising within social interactions.

From a theoretical standpoint, Social Cognitive Theory offers a useful framework for understanding how interpersonal environments influence eating behaviors, not only through direct behavioral modeling but also through emotional mechanisms. According to SCT, behavior is shaped by reciprocal interactions among personal factors, environment, and behavior itself (12). Within social eating contexts, observational learning (e.g., modeling others’ eating), self-efficacy (confidence in regulating intake), and social feedback (approval or disapproval from others) operate jointly to shape emotional experiences and subsequent food choices. For instance, social feedback can evoke emotions such as embarrassment or pride, which in turn influence self-regulation during eating episodes. Emotional states—emerging in or shaped by interpersonal interactions—can function as internal drivers or regulators of eating. Negative emotions such as anxiety, loneliness, or frustration may trigger maladaptive eating patterns, including emotional eating and binge eating (13, 14), while positive social emotions can promote adaptive self-regulation and enjoyment (15, 16). In line with emotion regulation theory (17), these processes suggest that individuals regulate eating behaviors partly to modulate affective states induced by social interaction. Thus, the pathway “social context – motional reaction – eating behaviors” reflects an integrated socio-emotional regulation loop.

Despite this growing recognition of the emotion–eating link, much of the existing literature remains constrained by laboratory paradigms that isolate emotion from real-world social interactions (18). These studies often induce emotions experimentally and capture short-term changes in food intake, leaving unanswered how emotional experiences naturally unfold and are expressed in authentic interpersonal settings. Recent advances in text mining and natural language processing have enabled psychologists to infer emotions, personality traits, and well-being from linguistic data (19). These computational approaches make it possible to analyze large-scale, naturally occurring language and to capture subtle emotional dynamics that are difficult to observe in laboratory settings. However, few studies have applied such methods to examine how social emotions expressed in language relate to behavioral regulation, particularly in the domain of eating. With the rise of digital communication, social media platforms such as Weibo provide ecologically valid opportunities to examine how individuals spontaneously express and regulate social emotions. Unlike traditional experiments, these platforms capture the linguistic traces of genuine emotional experience in social life (20). A growing body of research has examined how social media use relates to psychological well-being, showing both benefits (social connection, emotional support) and risks (social comparison, body dissatisfaction) (21; 22). However, few studies have integrated these insights to explore how social emotions expressed online relate to eating behaviors.

Building on these perspectives, the present study investigates how emotional experiences within social contexts are reflected in language and how such emotional expression relates to individual differences in eating behaviors. Specifically, we address two core questions: (1) What psychological and interpersonal themes emerge in naturally occurring discussions that link eating behaviors to social experience? (2) How do emotions expressed in social interactions relate to self-regulatory patterns in eating? To address these questions, we adopt a two-study design that integrates large-scale social media text mining with individual-level survey data, allowing us to link online discourse with eating behaviors. Study 1 explores the thematic structure and emotional patterns of social–dietary discussions on social media, providing a macro-level understanding of how social emotions are embedded in collective language use. Building upon these insights, Study 2 further examines, at the individual level, how social emotions influence intuitive eating behaviors through validated psychometric assessment and linguistic analysis.

## 2 Methods

### 2.1 Data

The dataset for Study 1 was obtained from Sina Weibo, one of China’s most influential social media platforms, which hosts large volumes of user-generated content reflecting daily life interactions and thus provides ecologically valid naturalistic language data for examining social behaviors and affective states (23). Using a customized web crawler, 3028 publicly accessible posts containing the keywords *“dietary behaviors”* and *“social interactions”* were collected. All retrieved posts were drawn solely from publicly available content that users had made openly accessible on the platform. No private messages or restricted-access materials were included. To ensure privacy protection, only anonymized textual content and non-identifiable metadata were retained, in accordance with ethical standards for secondary analysis of public online data (24). Metadata included user pseudonyms, post texts, geolocation tags (country/province/city), and device types. To maximize data coverage, no *a priori* temporal restrictions were imposed; instead, posts were continuously collected until June 2024, which served as the data collection endpoint. After data cleaning procedures (removal of duplicates, advertisements, and irrelevant content) and standard text preprocessing (tokenization and stopword removal), 1902 valid posts were retained for analysis.

Study 2 employed an online questionnaire to collect both textual data and psychometric assessments. First, participants’ recent social experiences were elicited through open-ended questions requiring detailed descriptions of interpersonal interactions. Second, intuitive eating behaviors were assessed using the validated Chinese version of the Intuitive Eating Scale–2. A total of 1,314 adult volunteers were recruited via social media platforms. After rigorous quality control to exclude invalid responses (e.g., patterned or contradictory answers, completion time <120 seconds), 1,199 valid responses were retained. The final sample comprised individuals aged 18–33 years (*M* = 21.16, *SD* = 2.14), including 798 females and 401 males. All participants provided informed consent, and the study was conducted in accordance with institutional ethical review guidelines.

### 2.2 Measurement

Textual data were obtained from Weibo using a customized Python-based web crawler developed by the research team. The program was designed to systematically capture user-generated content while minimizing data loss and redundancy. This approach facilitates reliable and reproducible acquisition of naturalistic social media texts, which are well-suited for examining social and affective processes in everyday contexts.

Participants’ eating behaviors were assessed using the validated Chinese version of the Intuitive Eating Scale–2 (IES–2; 25). The Chinese adaptation was psychometrically validated in previous research, showing satisfactory reliability and construct validity across both college (26) and clinical samples (27), thus supporting its applicability in Chinese populations. The 23-item measure comprises four subscales: Unconditional Permission to Eat (6 items; α = .61), Eating for Physical Rather Than Emotional Reasons (8 items; α = .75), Reliance on Hunger and Satiety Cues (6 items; α = .78), and Body–Food Choice Congruence (3 items; α = .73). Items were rated on a 5-point Likert scale (1 = *strongly disagree*, 5 = *strongly agree*), with higher scores indicating stronger intuitive eating tendencies (25). The subscales demonstrated acceptable internal consistency in the present sample.

Although the internal consistency of the “Unconditional Permission to Eat” subscale (α = .61) was lower than that reported in previous Chinese validation studies (α = .888–.919; 27), several contextual factors may account for this difference. First, our sample consisted of young adults from a non-clinical population, whose eating behaviors and self-perception of food restraint may be less stable than those of clinical or patient groups. Second, the items of this subscale are particularly sensitive to social and cultural norms around eating restraint, which may lead to greater within-group variability and reduced internal consistency in community samples. Given its theoretical relevance in representing a core aspect of intuitive eating, the subscale was retained for analysis. Nevertheless, the lower internal consistency may have introduced greater measurement error and attenuated observed associations between this dimension and other variables. Therefore, interpretations involving this subscale should be made with caution, and future research is encouraged to further refine its linguistic adaptation for diverse Chinese populations.

### 2.3 Analytical methods

Topic modeling is an effective method for identifying and organizing latent themes within large-scale text data (28). Among these methods, Latent Dirichlet Allocation (LDA) assumes that each document is composed of multiple latent topics, and each topic is characterized by a distinct word distribution (29). A key challenge in applying LDA lies in determining the optimal number of topics (30). An excessive number of topics may cause overfitting, whereas too few topics may oversimplify the text and obscure meaningful distinctions (31)To address this issue, the present study employed a perplexity-based optimization procedure, where lower perplexity values indicate a better-fitting model (32–34). The calculation of perplexity is shown in Equation 1.

(1) $$perplexity\left(D_{test}\right)=exp\left\{-\frac{\sum_{d=1}^{M}\log p\left(w_{d}\right)}{\sum_{d=1}^{M}N_{d}}\right\}$$

Here: $p(w_{d})\;$ denotes the ​generative probability of document *d.* $N_{d}\;$ represents the ​total number of words in document *d*.

To further process the text data, we employed semantic network analysis, a technique that reveals language structure by analyzing co-occurrence relationships between words (35). This method treats words or phrases as nodes and their co-occurrence as edges, constructing a semantic network for visualizing and quantifying underlying semantic structures. In this study, Pointwise Mutual Information (PMI; 36) was used as a key metric to measure the statistical association between word pairs. PMI effectively reduces the influence of high-frequency words while highlighting low-frequency but semantically significant word pairs. A higher PMI value indicates a stronger co-occurrence relationship. The definition of PMI is given in Equation 2.

(2) $$\begin{array}{c} PMI(x,y)=\log\frac{P(x,y)}{P(x)P(y)} \end{array}$$

Here: P(x,y) is the probability of the two words co-occurring, while P(x) and P(y) represent the individual probabilities of each word occurring.

LASSO (Least Absolute Shrinkage and Selection Operator) regression is a statistical method that performs ​simultaneous variable selection and model shrinkage by imposing an $L_{1}$​-norm penalty on regression coefficients (37). The regularization parameter λ, a critical hyperparameter in LASSO, governs the intensity of the penalty term and directly modulates the ​sparsity structure of the model. Optimal λ selection is typically implemented via ​cross-validation, which supports a balance between bias and variance and promotes generalization performance.

### 2.4 Analysis procedure

**Figure 1:**
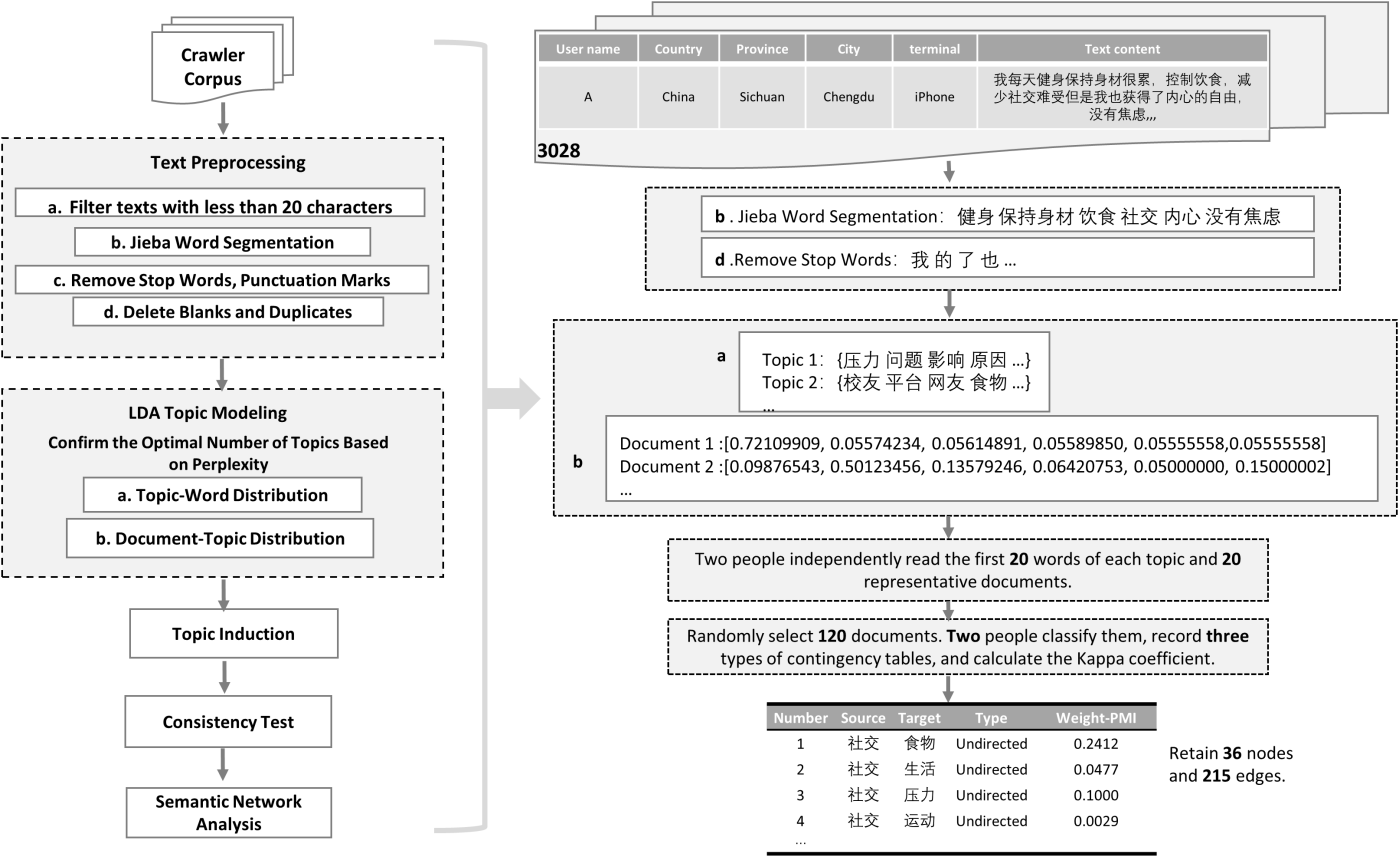
Flowchart of the data processing steps in Study 1.

Study 1 was designed to identify core discourse themes surrounding “dietary behaviors” and “social interactions” on social media platforms and explore their latent associations with users’ mental health, affective states, and social engagement patterns. To achieve these objectives, we implemented a five-stage analytical pipeline (see **Figure 1**), structured as follows:

1. Corpus Standardization: To ensure data quality, raw textual data underwent rigorous preprocessing. Posts containing fewer than 20 characters were excluded to preserve semantic validity. Remaining texts were tokenized using Python’s Jieba library, followed by removal of stopwords, punctuation, whitespace, and duplicate entries, yielding a structured analytical corpus.
2. Latent Thematic Extraction: The LDA model was constructed using the *LatentDirichletAllocation* module in scikit-learn (Python). Model parameters were set as follows: α = 0.1 for the document–topic distribution and *β = 0.01* for the topic–word distribution. The model was trained using a batch learning approach (*learning_method=‘batch’*) for up to 100 iterations (*max_iter=100*), with a fixed random seed (*random_state=42*) to ensure reproducibility. The optimal number of topics was determined by jointly considering the lowest perplexity.
3. Dual-Coder Thematic Labeling: Two domain-trained graduate researchers independently derived theme labels by analyzing each theme’s top 20 keywords and 20 representative documents. Iterative discussions resolved coding discrepancies (38), culminating in consensus-based thematic nomenclature.
4. Classification Consistency Validation: Two independent coders annotated 120 randomly sampled posts (39, 40). Cohen’s Kappa coefficient (41) quantified inter-rater agreement between LDA outputs and manual annotations, objectively evaluating theme robustness.
5. PMI Network Analysis: To complement LDA’s semantic limitations, a co-occurrence network was constructed using the Pointwise Mutual Information (PMI) metric. Nodes were first selected based on word frequency (top 120 terms) and PMI values above the 75th percentile to ensure statistical relevance. Edge weights corresponded to PMI scores, and connections with weights below 0.1 were excluded to enhance interpretability. Community detection was conducted using the Louvain modularity optimization algorithm implemented in Gephi, which automatically classified nodes into modularity classes representing distinct semantic communities (42). Weighted degree was computed for each node to quantify its centrality within the network. Following this, both network metrics (e.g., modularity, degree centrality) and semantic coherence were jointly considered to retain 36 representative nodes and 215 statistically significant edges for final visualization.

**Figure 2:**
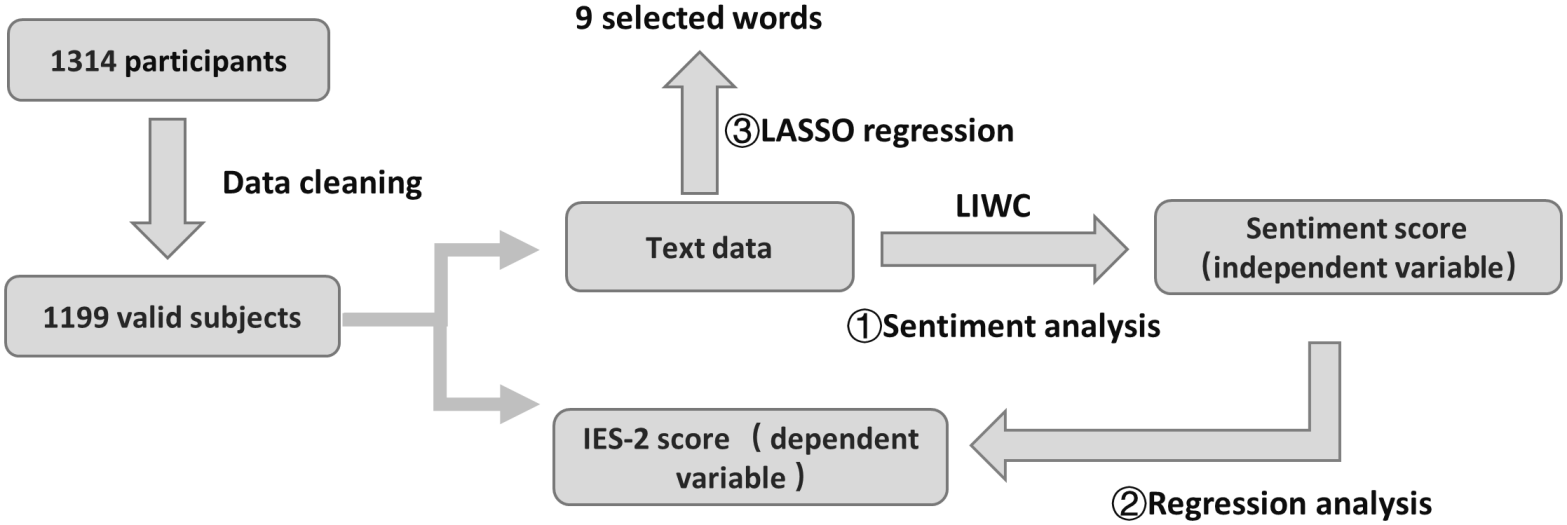
Schematic diagram of the data processing flow in Study 2.

Building upon the emotional and thematic patterns identified in Study 1—particularly those highlighting the interplay between social emotions, stress, and dietary behaviors—Study 2 extends this line of inquiry to an individual behavioral level. Specifically, Study 2 systematically investigates the mechanistic links between social emotional states and intuitive eating behaviors, while identifying semantically salient markers associated with eating patterns through advanced text feature extraction. The three-stage analytical protocol (see **Figure 2**) unfolds as follows:

1. Affective Profiling via LIWC Lexicon: To quantify emotion-laden expressions in social discourse, we deployed the Linguistic Inquiry and Word Count (LIWC-22) software for automated sentiment annotation. This tool calculates standardized scores for ​positive affect (e.g., “joy,” “satisfaction”) and ​negative affect (e.g., “anxiety,” “loneliness”) by matching user-generated posts against its empirically validated emotion lexicon, establishing a quantifiable foundation for linking affective dimensions to intuitive eating outcomes.
2. Multivariate Regression Modeling: A multiple linear regression model was implemented in SPSS 27 to assess the predictive validity of emotional variables. Specifically, LIWC-derived positive and negative affect scores served as core predictors, with the total score from the Intuitive Eating Scale-2 (IES-2) as the dependent variable. This approach disentangled the differential predictive strength of distinct emotional domains on eating behaviors.
3. LASSO Regression for Semantic Marker Identification: To transcend limitations of conventional regression, machine learning-enhanced text mining was conducted: Python’s scikit-learn TfidfVectorizer converted raw texts into Term Frequency-Inverse Document Frequency (TF-IDF) matrices, prioritizing high-frequency lexical candidates; using the glmnet package in R, LASSO regression (43) was applied with IES-2 scores as the response variable and TF-IDF features as predictors. Optimal regularization parameters were selected via 10-fold cross-validation, retaining non-zero coefficient terms to pinpoint semantically critical markers of intuitive eating behaviors.

## 3 Result

### 3.1 Topic analysis

**Figure 3:**
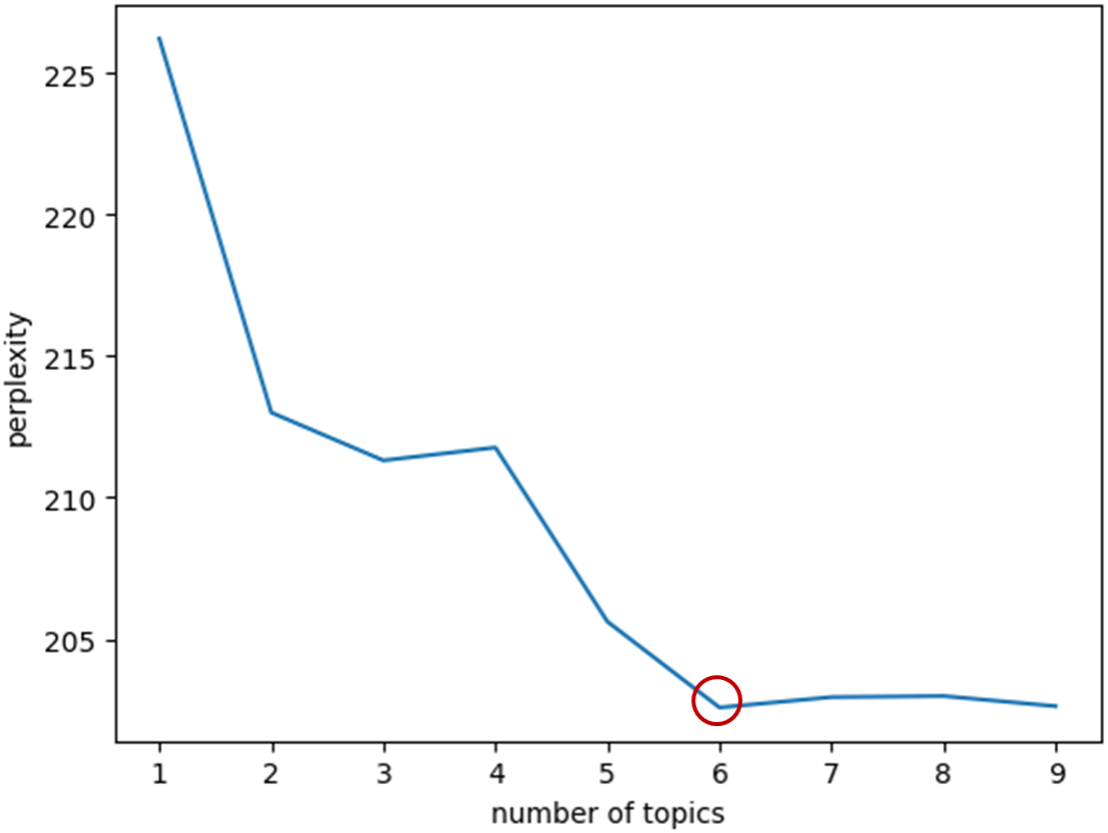
Trend of perplexity with varying number of topics.

The optimal number of topics and thematic extraction results are illustrated in **Figure 3** and **Table 1**. Based on the LDA model, the lowest perplexity was achieved when the number of topics was set to 6, indicating superior model fit under this configuration (29). The six extracted topics are as follows: ​Life Stress and Emotional Management, Gourmet Experiences and Media Interaction, Mental Health and Stress, Alumni Gatherings and Socialization, Social Anxiety and Image Management, Psychological States and Exercise Regulation. Each theme was labeled not only based on lexical co-occurrence patterns but also with reference to its underlying psychological meaning—for example, “Life Stress and Emotional Management” aligns with Lazarus’s stress and coping framework, while “Social Anxiety and Image Management” reflects self-presentation and social comparison processes. The top 20 keywords and corresponding document counts for each topic reflect users’ focal concerns and emerging trends in discussions related to social interactions and dietary behaviors.

**Table 1:** Summary of thematic topics with top keywords and document frequencies from LDA topic modeling.

No.	Topic	The top 20 keywords of topic	Number of documents
1	​Life Stress and Emotional Management	life, work, stress, exercise, food, body, time, way/method, routine, learn, relationship, energy, feeling, mental/spirit, friends, emotion, human, management, people, daily schedule	501
2	Food Experiences and Media Interaction	food, media, delicacies, friends, video, oral, bad breath, hotpot, distance, communication, pandemic, mobile phone, information, platform, world, software, game, teacher, unable, experience	409
3	Mental Health and Stress	stress, problem, influence/impact, cause, children, research, emotion, environment, plan, social activities, disease, exercise, patients, disorder, goal, aspect, symptoms, nutrition, doctor, population	269
4	Alumni Gatherings and Socialization	alumni, platform, netizens, food, proposal, gathering/dinner, anniversary, grandma, activity, restaurant, photos, entire group, dining, high-speed rail, news, greasy, go viral, group, internet, stomach	140
5	Social Anxiety and Image Management	social anxiety/social phobia, weight, feeling, exercise, friends, body shape, a bit, record, go home, gym, meaning, clothes, smile, time, single, place, food, street, diary, video	367
6	Psychological States and Exercise Regulation	state, things/matters, stress, psychology, routine, exercise, body, schedule, reasonable, emotion, mindset, means, constructive, school starts, video, mobile phone, inner world, self-discipline, guideline, skin	216

**Figure 4:**
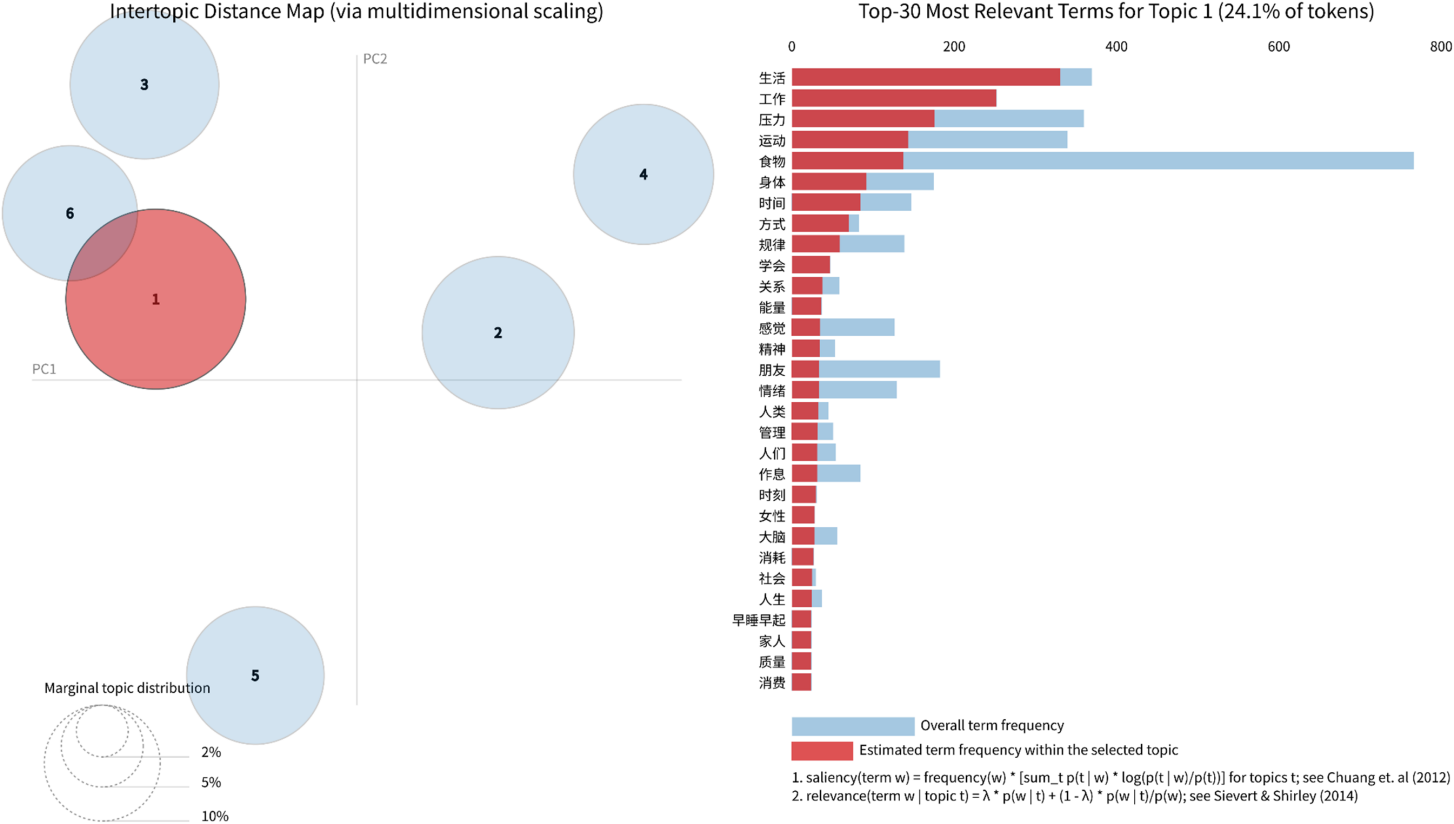
Topic modeling visualizations generated with pyLDAvis in Python, showing the relationship between the number of topics and perplexity, inter-topic distances, marginal topic distribution, and the top 30 relevant terms (using Topic 3 as an example).

Multidimensional Scaling (MDS) analysis, implemented via the pyLDAvis package, visually represents the inter-topic relationships (44). The visualization of topic spatial distributions is depicted in **Figure 4**, where bubble labels correspond to topic indices, and bubble diameter scales proportionally to the document count within each topic. Taking Topic 1 as an exemplar, the right panel of **Figure 4** displays the 30 most relevant terms for this topic, along with their proportional representation among all tokens (24.1%). (Detailed visualizations for other topics are provided in **Supplementary Material 1**) Inter-bubble distances reflect semantic associations between topics: proximate bubbles indicate thematic overlaps, while distant ones suggest content independence.

The consistency check of topic classification revealed that the Cohen’s Kappa coefficients between the LDA model and Researcher A and Researcher B were 0.470 and 0.450, respectively (both *p* <.001), while the coefficient between Researcher A and Researcher B was 0.504 (*p* <.001). These values fall within the range of moderate agreement (45) and are comparable to those typically reported in topic modeling or mixed-method semantic analyses (39), where κ values between 0.40 and 0.60 are generally considered acceptable given the interpretive nature of theme labeling (39, 46).This level of consistency may be attributed to the semantic multiplicity of the texts—for instance, a term like “stress relief” could be categorized under both Topic 1 (“Life Stress and Emotional Management”) and Topic 3 (“Mental Health and Stress”) due to its contextual relevance. Such overlap reflects the inherent ambiguity of user-generated content, in which emotional and behavioral expressions often span multiple domains. Future refinements could involve adopting hierarchical topic models or incorporating word embeddings (Word2Vec, BERT) to capture semantic nuances more precisely and reduce coder uncertainty.

### 3.2 Semantic network analysis

**Figure 5:**
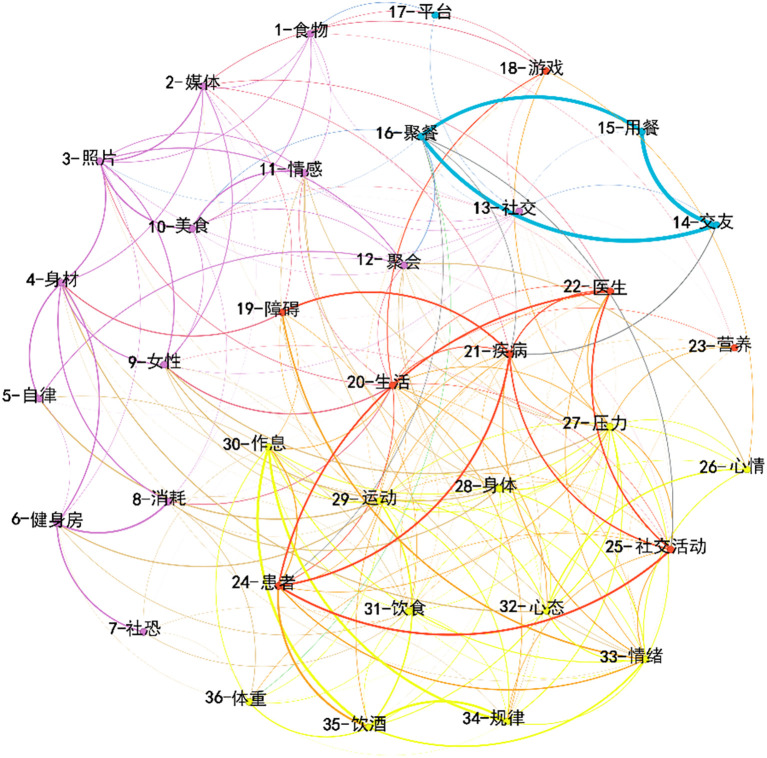
Semantic network analysis of Weibo discussions related to social and dietary themes. The nodes in the picture are as follows: 1 - Food, 2 - Media, 3 - Photo, 4 - Body figure, 5 - Self - discipline, 6 - Gym, 7 - Social phobia, 8 - Consumption, 9 - Female, 10 - Delicacy, 11 - Emotion, 12 - Gathering, 13 - Social interaction, 14 - Make friends, 15 - Dine, 16 - Dinner party, 17 - Platform, 18 - Game, 19 - Obstacle, 20 - Life, 21 - Disease, 22 - Doctor, 23 - Nutrition, 24 - Patient, 25 - Social activity, 26 - Mood, 27 - Stress, 28 - Body, 29 - Exercise, 30 - Work and rest schedule, 31 - Diet, 32 - Mentality, 33 - Emotion, 34 - Regularity, 35 - Drink alcohol, 36 - Weight.

The network, comprising 36 key nodes and 215 edges, exhibited an average degree of 41.87 and a mean weighted degree of 49.19, indicating a relatively dense interconnection among high-frequency terms (network density = 0.341). The modularity coefficient (Q = 0.28) suggested a moderately clustered semantic structure, reflecting that while distinct communities emerged, substantial overlaps existed across social and dietary themes—a pattern typical of psychologically intertwined discourse. It was divided into four primary categories using modular processing, with each category visually distinguished by color, as shown in **Figure 5**. The blue nodes represent social-dietary interactions, highlighting the connection between food choices and social contexts, such as gatherings with friends and festive celebrations. The red nodes focus on health-related discussions, reflecting the role of social media in facilitating the exchange of health information, including topics on diseases, lifestyle habits, and dietary recommendations. The yellow nodes illustrate emotion-physiology linkages, emphasizing the interplay among diet, emotional regulation, physical activity, and weight management, encompassing aspects like stress relief, mood regulation, and healthy living practices. Lastly, the purple nodes center on body image–social anxiety, examining the relationship between social media use, self-perception, and the interplay between social anxiety and eating behaviors.

### 3.3 Key predictive word analysis based on LASSO regression

Building upon the thematic and semantic findings in Study 1, Study 2 further explored how social-emotional expressions in interpersonal contexts relate to individual eating behaviors. Based on social text data from 1,199 valid participants, the study first quantified emotional indicators through sentiment analysis and conducted multiple linear regression analyses using the LIWC emotion scores and the total score of the Intuitive Eating Scale-2 (IES-2). As shown in **Table 2**, the negative emotion score in social texts was significantly negatively associated with intuitive eating behaviors (*β* = -0.103, *p* = 0.023), indicating that individuals who express stronger negative emotions in social interactions are more likely to experience impaired intuitive eating abilities.

**Table 2:** Regression coefficients of social emotional scores predicting intuitive eating.

Model	Unstandardized coefficient B	Standardized coefficient Beta	t	P
constant	77.903		158.378	0.000
Social- positive	0.018	0.020	0.665	0.506
Social- negative	-0.103	-0.067	-2.271	**0.023**

*Bold values indicate statistical significance (p < 0.05).

**Figure 6:**
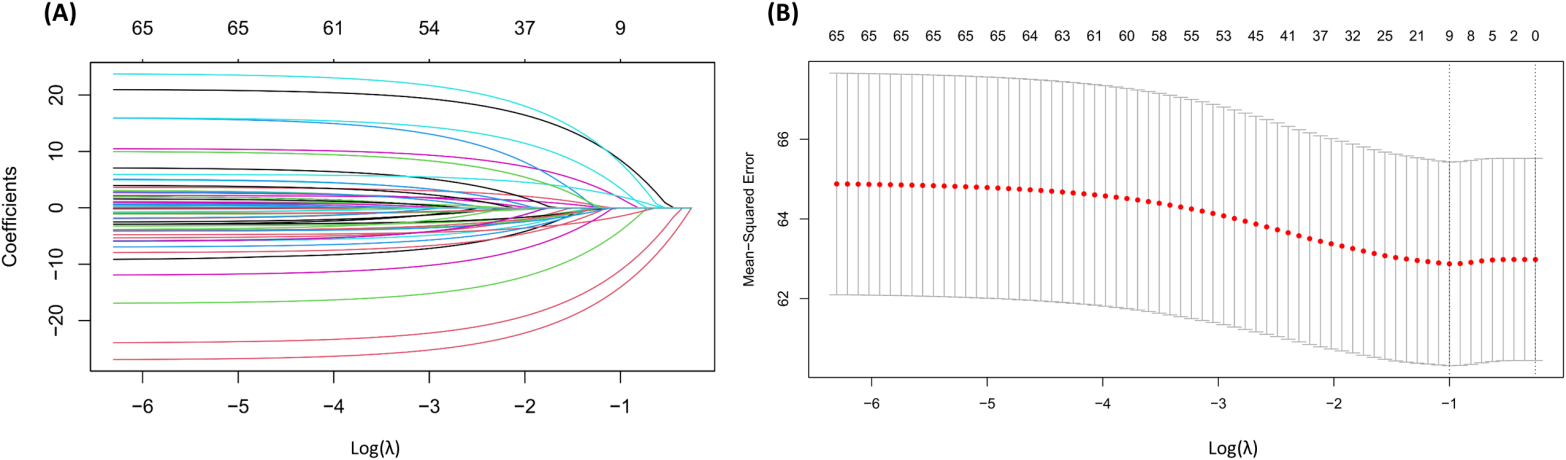
Visualization of the effects of log(λ) on LASSO regression. **(A)** LASSO regression coefficients. **(B)** Mean squared error (MSE) across different λ values.

To further identify key semantic markers related to eating behaviors, the study employed LASSO regression analysis using the glmnet package in R. LASSO regression introduces an $L_{1}$​-norm penalty, which gradually shrinks the coefficients of less important variables to zero. The LASSO regression model yielded a cross-validated mean squared error (MSE) of 62.87 with an R² of 0.08. While the explained variance is modest, this level of performance is expected given the high-dimensional nature of linguistic data. In this context, the primary objective of LASSO regression was not to maximize predictive accuracy, but to identify linguistic features most strongly associated with intuitive eating scores. This feature selection approach aligns with established practices in psychological text-mining research, where interpretability and parsimony take precedence over raw prediction performance (47, 48). As the penalty parameter λ increases, most coefficients converge toward zero (as shown in **Figure 6**). The optimal regularization parameter λ was determined through cross-validation, ultimately retaining nine non-zero coefficient terms (as presented in **Table 3**). Among them, “sensitive,” “tiring,” “frustrated,” and “lonely” were negatively associated with intuitive eating scores, suggesting that expressions of these emotions may impair individuals’ self-regulation of eating. In contrast, “bored,” “anxious,” “shy,” “positive,” and “relaxed” demonstrated positively associated with intuitive eating scores, implying that moderate anxiety and a positive mindset may enhance cognitive control and promote healthier dietary behaviors. Notably, the unexpected positive associations of traditionally negative words like “bored”, “anxious” and “shy” may reflect individuals’ adaptive strategies of using dietary management to cope with social stress.

**Table 3:** Key emotional words predicting intuitive eating scores.

Number	Word	Cofficient
constant term		77.809
1	Sensitive	-14.078
2	Tiring	-11.078
3	Frustrated	-4.078
4	Lonely	-1.493
5	Bored	8.460
6	Anxious	7.953
7	Shy	3.460
8	Positive	2.005
9	Relaxed	1.693

Across the three analytical approaches, a convergent pattern emerged linking emotional experiences in social contexts with dietary behaviors. The topic modeling results revealed that users frequently discussed stress, emotional regulation, and body image concerns in relation to food and social interaction. The semantic network analysis further confirmed these interconnections, demonstrating that emotional, social, and dietary terms were densely intertwined and clustered around shared psychological constructs such as stress management and self-presentation. Extending these findings to individual-level data, the linguistic analysis identified emotion-related expressions that covaried with intuitive eating tendencies. Taken together, these results provide multi-level evidence that social-emotional dynamics play a central role in shaping both the discourse and behavioral dimensions of eating.

## 4 Discussion

The present study provides novel insights into the psychological mechanisms linking social emotions and eating behaviors by integrating large-scale social media analysis with individual-level assessments. The findings reveal associative patterns between emotional expression in social contexts and self-regulatory aspects of eating. By combining topic modeling, semantic network analysis, and behavioral validation of emotion-related language, this research advances a multi-method framework for understanding how social-emotional processes are reflected in dietary discourse and behavior.

The structured analysis of six thematic categories indicated that emotional states frequently co-occur with eating-related discussions. The clustering of psychological themes—”Mental Health and Stress,” “Life Stress and Emotion Management,” and “Psychological States and Exercise Regulation”—illustrates that stress and emotion regulation are recurrent themes in public discourse surrounding diet. These findings are consistent with prior evidence that stress correlates with reduced healthy food intake and greater consumption of high-calorie foods (49, 50). Extending this evidence base, the present results suggest that such emotional dynamics are traceable within naturally occurring online language, highlighting the value of digital data for psychological inquiry.

Study 2 further supported this mechanism by showing that negative emotions expressed in social texts were significantly associated with lower intuitive eating. Words such as “tiring,” “frustrated,” and “lonely” were associated with weaker dietary self-regulation, echoing prior work linking negative affect and maladaptive eating behaviors (51–53). This convergence of online linguistic signals and offline self-reports underscores the reliability of emotion–diet associations across methodological contexts. Conversely, positive expressions such as “relaxed” and “positive” were linked to healthier eating behaviors, suggesting that supportive emotional climates can buffer against maladaptive eating patterns. Interestingly, certain words often coded as negative (“bored,” “anxious,” “shy”) showed positive associations with intuitive eating, highlighting the complexity of emotional regulation. This pattern may reflect a higher level of emotional awareness, in which individuals recognize and articulate their transient discomfort rather than avoiding it (54). From the perspective of reflective self-regulation, acknowledging mild social unease (e.g., boredom or anxiety) can prompt individuals to monitor internal states more deliberately, thereby engaging in more conscious food-related decisions (55). In this sense, such emotions may function as regulatory cues rather than risk factors, signaling opportunities for self-reflection and adaptive adjustment to eating behaviors. These results suggest that not all negative emotions are uniformly maladaptive, some may heighten self-awareness and promote more deliberate food choices.

Beyond individual emotional states, the results emphasize the role of social environments and body image concerns in shaping eating regulation. The themes “Food Experiences and Media Interaction” and “Alumni Gatherings and Socialization” illustrate that both online sharing and offline social gatherings are associated with eating choices. While the mechanisms cannot be directly inferred from the topic modeling, prior research suggests that self-presentation, peer influence, and normative expectations are likely drivers of these associations (6, 8, 56). In particular, the relative isolation of the “Social Anxiety and Image Management” theme points to a distinct pathway, which may reflect a tendency toward restrictive eating as a coping strategy to manage social evaluation risks. This interpretation aligns with Social Identity Theory (57), which posits that individuals regulate their behaviors to maintain a favorable social identity and group acceptance, and with the Social Media Internalization Model (58), which highlights how internalized appearance norms derived from digital comparison processes shape body-related attitudes and eating behaviors. Taken together, these findings suggest that dietary regulation in social contexts is not only an outcome of individual emotional control but also reflects collective identity dynamics and social conformity pressures within both online and offline environments.

While the psychological findings resonate with existing health behavior research (59–61), While the psychological findings resonate with existing health behavior research (59–61), this study makes a distinctive contribution by situating these mechanisms within the context of digital platforms. The semantic network analysis showed that health-related nodes (“Doctor,” “Disease,” “Patient”) coexist with social nodes (“Dining,” “Gathering”) and emotional nodes (“Stress,” “Body”), reflecting how health communication on social media is deeply interwoven with social bonding and emotional states. This hybrid structure can be better understood through the lens of social cognitive theory (62) and the social sharing of emotion framework (63). From a social cognitive perspective, online environments provide both observational learning cues and social reinforcement that shape individuals’ health-related behaviors. Users not only receive information about diet or exercise but also observe emotional expressions and social feedback that reinforce certain attitudes or practices. Meanwhile, the social sharing of emotion framework suggests that emotional disclosure—such as expressing stress or body concerns—serves to regulate affect and strengthen social bonds. The co-occurrence of emotional and health-related terms in the network thus reflects a collective coping mechanism, where individuals use social media to negotiate both personal well-being and social belonging.

From a platform perspective, the identification of emotion-laden linguistic patterns related to eating behaviors suggests possible directions rather than direct interventions for supporting users’ emotional and dietary well-being. For example, digital health applications could be informed by digital behavior change intervention (DBCI) principles to help users recognize emotional stress and adopt healthier coping strategies. At the design level, platforms might explore ways to reduce content that reinforces body anxiety and to promote balanced, positive discussions about food and well-being. These directions fit within broader digital mental health frameworks, which emphasize supportive online environments and self-awareness rather than behavioral control (64).

Beyond its practical relevance for digital health design, the present findings also carry theoretical significance. First, it applies and refines Social Cognitive Theory (12) within digital contexts, demonstrating that observational learning and social reinforcement mechanisms are not limited to offline interactions but are also salient in online environments, where peer influence, self-presentation, and **s**ocial comparison processes are intensified (65, 66). This extension underscores how digital platforms serve as social learning environments that both mirror and magnify everyday behavioral modeling. Second, the study provides evidence that natural language on social media can serve as an observable marker of underlying psychological and emotional states. This aligns with emerging perspectives in emotional awareness (67) and reflective self-regulation frameworks, which posit that individuals’ capacity to articulate emotions linguistically reflects deeper levels of self-monitoring and behavioral control. By linking online emotional expression to offline eating behaviors, this work advances the integration of affective and behavioral dimensions in health psychology. Third, the research offers a methodological demonstration of integrating big data analytics (LDA topic modeling and semantic network analysis) with behavioral assessment. This framework highlights how computational methods can complement psychological theory to capture complex emotion–behavior dynamics in naturalistic digital environments.

Despite these contributions, several limitations must be acknowledged. First, the data from Study 1 were collected solely from Weibo, a Chinese platform, which may limit the generalizability of findings to other contexts. Cultural norms around emotional expression and eating behavior may differ substantially across societies, particularly between collectivist and individualist cultures. Future research should examine cross-cultural comparisons across platforms such as Twitter, Instagram, or TikTok. Second, although LDA and LASSO offer powerful tools for identifying consistent associations between emotional expression and eating behaviors, these methods do not imply causality. The observed relationships should therefore be understood as correlational patterns rather than causal effects. To determine whether changes in emotional expression directly influence eating regulation, more rigorous longitudinal designs will be necessary. Third, the reliance on text data overlooks multimodal expressions—images and videos of food, exercise, and body image are highly salient in social media contexts and likely shape eating behaviors in ways not captured by textual analysis. Future studies should incorporate multimodal approaches using computer vision and multimodal deep learning models. Finally, although this study applied dictionary-based approaches for emotion detection, more advanced natural language processing models (e.g., BERT) could capture contextual nuances in emotion expression, improving cross-cultural applicability.

## 5 Conclusion

This study provides evidence that emotional expression in social contexts—both online and offline—is associated with young adults’ eating behaviors. By integrating large-scale social media analyses with individual-level assessments, the research highlights potential pathways through which social emotions may relate to eating regulation. The topic modeling and semantic network results shed light on the thematic structures linking diet, emotion, and social interaction, while linguistic patterns identified through LASSO regression suggest specific emotional expressions associated with intuitive eating. Collectively, these findings enrich theoretical understanding of the psychosocial processes connecting emotion, social interaction, and eating, and offer practical implications for fostering healthier digital environments. While these findings highlight meaningful associations between social emotion and eating behaviors, future research is needed to clarify the causal mechanisms underlying these links.

## Data Availability

The raw data supporting the conclusion of this article is not publicly available a protect participant confidentiality and privacy. Requests to access the datasets should be directed to the corresponding author.
