# Supplementary material for: The relationship between social emotions and intuitive eating behaviors: an exploration based on text analysis
Source: Front Psychiatry. 2025 Nov 26;16:1701751. doi: 10.3389/fpsyt.2025.1701751 (PMC12690209; doi:10.3389/fpsyt.2025.1701751)
Supplement: Supplementary file 1 [file Image1.pdf]

Supplementary Material

Description: Supplementary Material 1. Visualizations of topic-word distributions for Topics 1-6, generated via pyLDAvis.

Topic 1 has already been presented in the main text; however, it is repeated in the supplementary materials to ensure overall completeness.

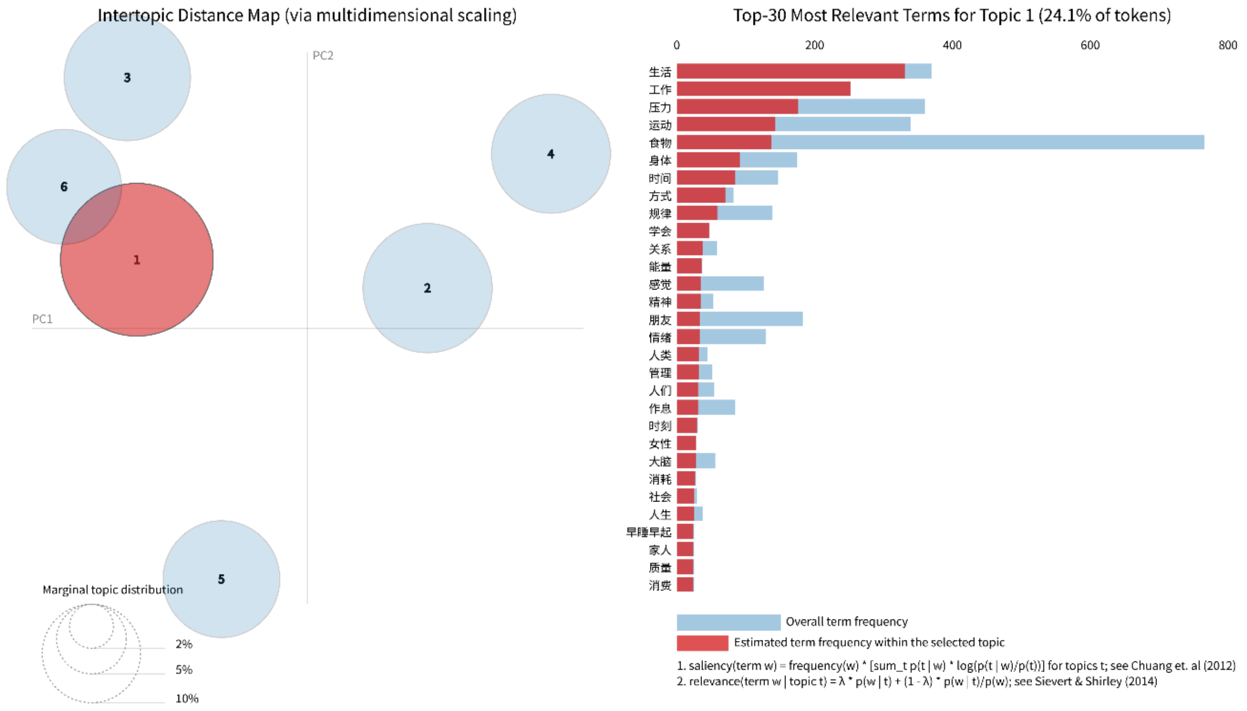

Supplementary Figure 1. Topic 1 Life Stress and Emotional Management

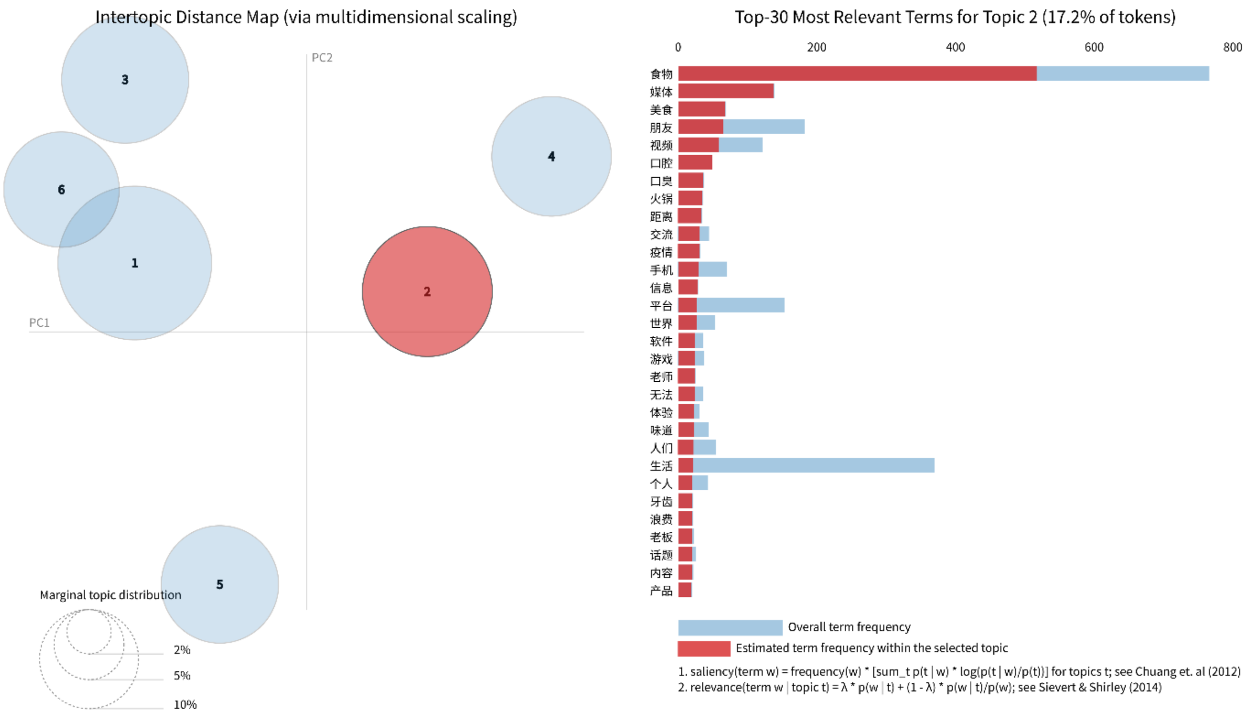

Supplementary Figure 2 Topic 2 Food Experiences and Media Interaction

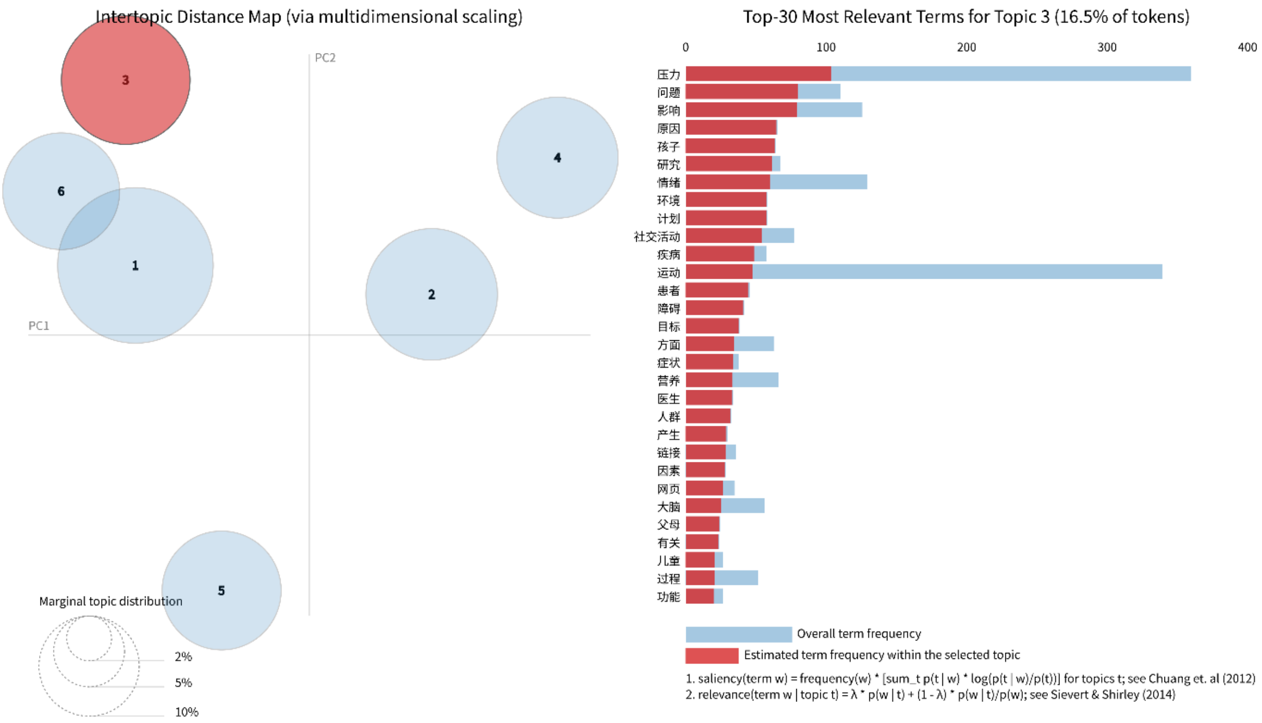

Supplementary Figure 3. Topic 3 Mental Health and Stress

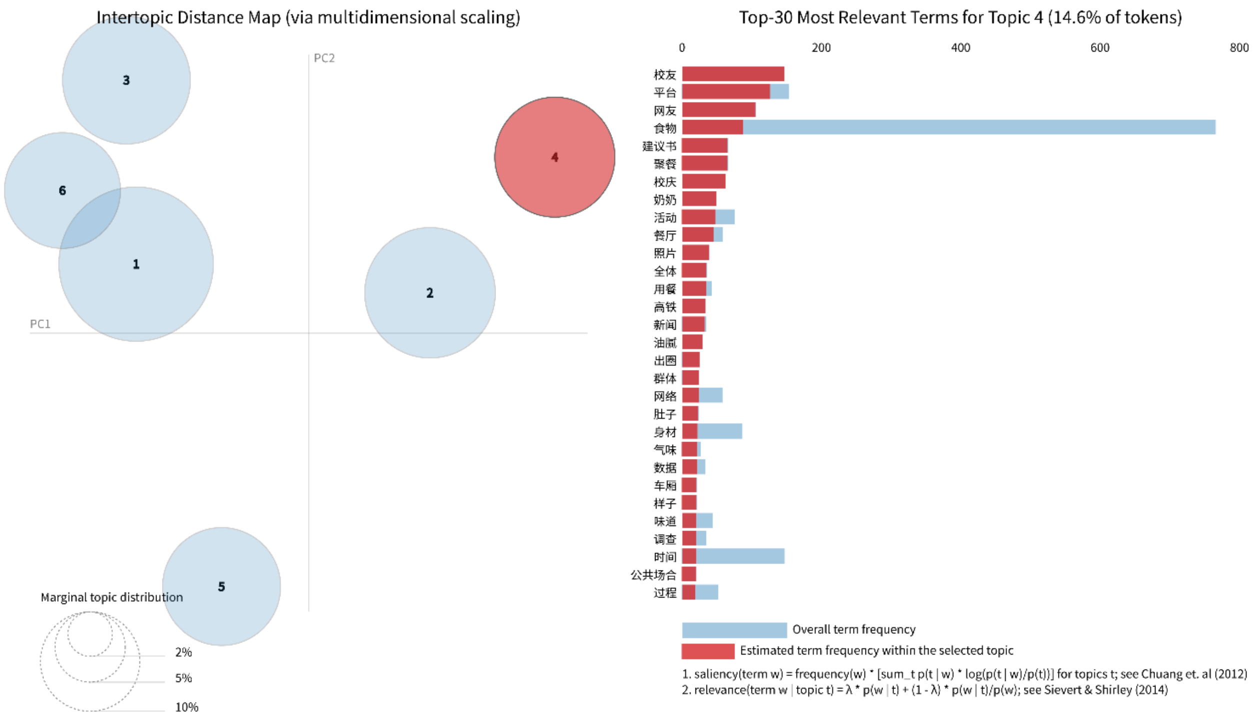

Supplementary Figure 4. Topic 4 Alumni Gatherings and Socialization

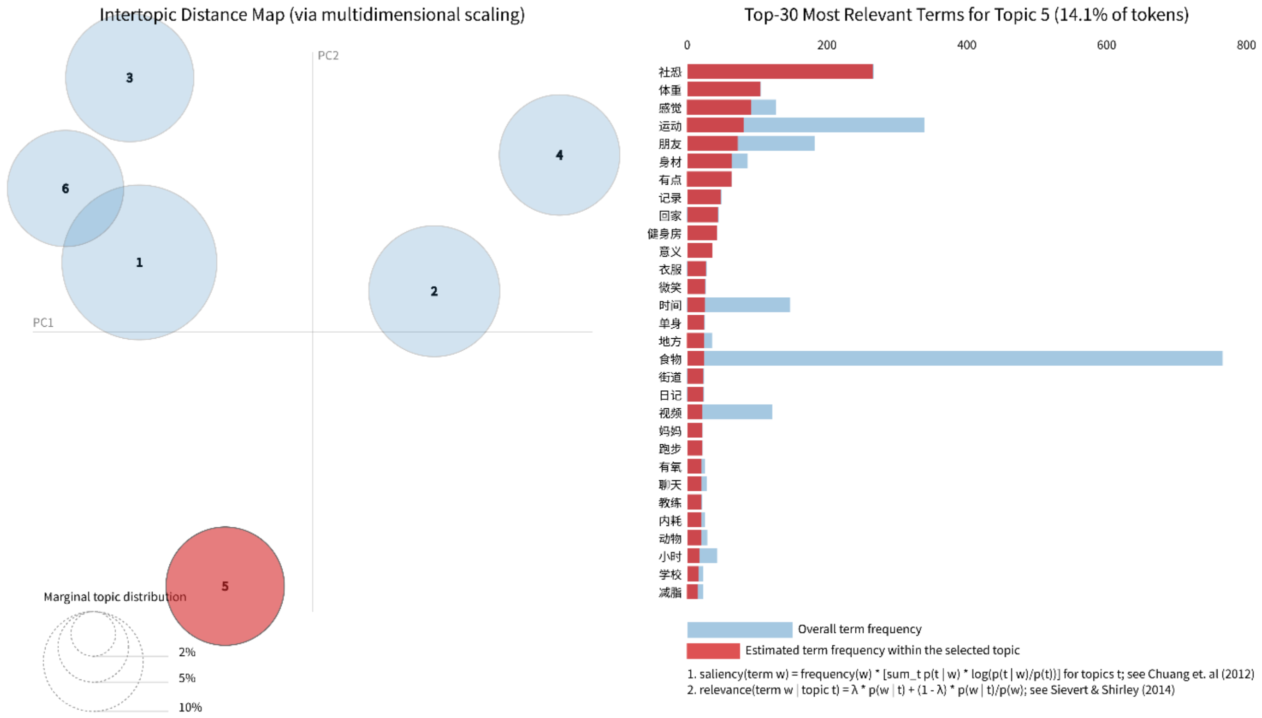

Supplementary Figure 5. Topic 5 Social Anxiety and Image Management

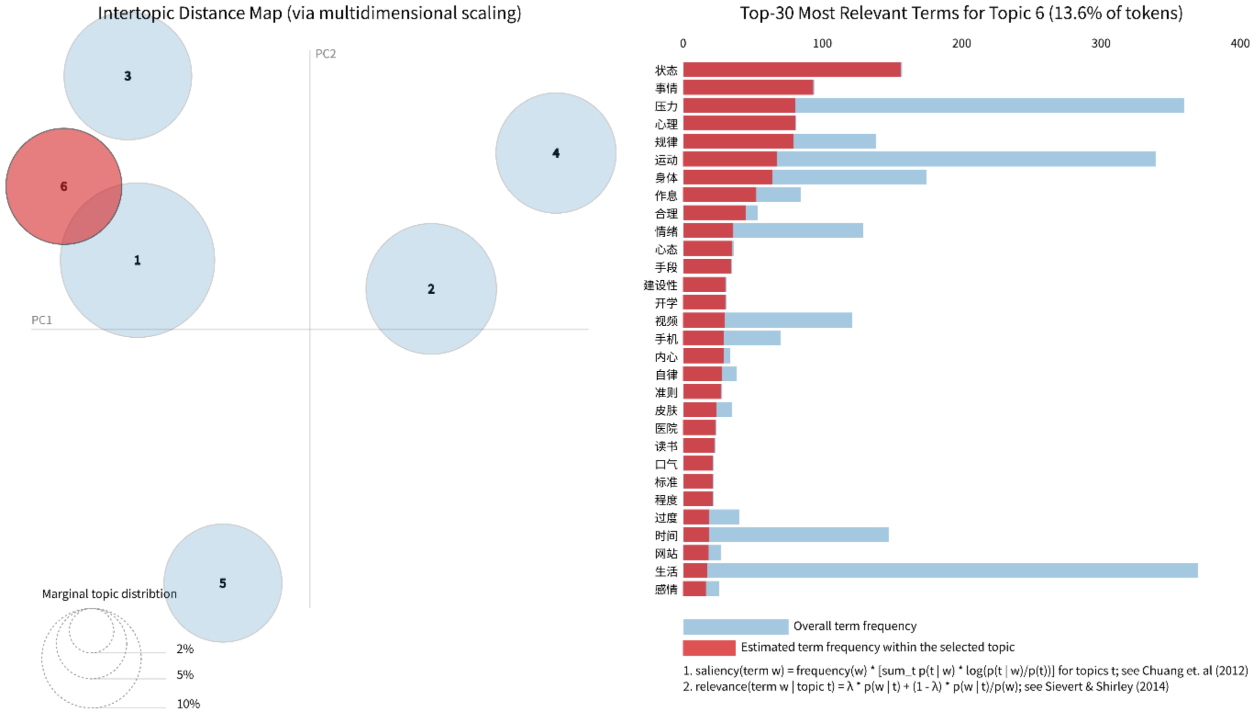

Supplementary Figure 6. Topic 6 Psychological States and Exercise Regulation
